# Design, Implementation and Simulation of a Fringing Field Capacitive Humidity Sensor

**DOI:** 10.3390/s20195644

**Published:** 2020-10-02

**Authors:** Adrian-Razvan Petre, Razvan Craciunescu, Octavian Fratu

**Affiliations:** Faculty of Electronics, Telecommunications and Information Technology, University Politehnica of Bucharest, 060042 București, Romania; razvan.petre@tensor.ro (A.-R.P.); razvan.craciunescu@upb.ro (R.C.)

**Keywords:** fringe-field, humidity sensor, capacitive measurement, low power, agriculture, IoT

## Abstract

The world population is growing in an accelerated way urging the need for a more efficient and sustainable agricultural industry. Initially developed for smart cities which face the same challenges caused by an increasing population, Internet of Things (IoT) technologies have evolved rapidly over the last few years and are now moving successfully to agriculture. Wireless Sensor Networks (WSNs) have been reported to be used in the agri-food sector and could answer the call for a more optimized agricultural management. This paper investigates a PCB-made interdigited capacitive (IDC) soil humidity sensor as a low-price alternative to the existing ones on the market. An in-depth comparative study is performed on 30 design variations, part of them also manufactured for further investigations. By measurements and simulations, the influence of the aspect ratio and dielectric thickness on the sensitivity and capacitance of the sensor are studied. In the end, a Humidity and Temperature Measurement Wireless Equipment (HTMWE) for IoT agriculture applications is implemented with this type of sensor.

## 1. Introduction

The world population reached 7.6 billion in 2017 and is expected to reach 8.6 billion in 2030, 9.8 billion in 2050 and 11.2 billion in 2100 according to a projection made by the Population Division of the United Nations Department of Economic and Social Affairs [1]. With the increase of world population new challenges rise, one of them being to provide enough food. Studies conducted in recent years have shown that the use of innovative technologies and a good management approach could improve irrigation scheduling, save water, and increase food productivity.

Zia et al. reviewed extensively outdated agriculture practices and proposed a Water Quality Monitoring, Control and Management (WQMCM) and an integrated framework that shares information among different networked farms [2]. Their work on the initial model was completed by concluding that applications of Wireless Sensors Network (WSN) hold a huge development potential for the agriculture sector [3,4]. Zia et al. also developed a predictive model for the nitrate loss, a source of pollution resulted from the conversion of nitrogen, using the same WQMCM framework [5].

Conventional agriculture was further investigated and resulted that it provides 40% of the world’s food from 20% of the agricultural land but uses 70% of all global freshwater. Rivers et al. stated that alternative irrigation systems outperform the conventional one, transforming the food issue into one of optimization with the help of smart interconnected devices in a WSN real-time reporting parameters from the soil [6]. WSNs have been widely adopted in the agriculture field due to the simplification in wiring resulted from their use [7,8].

A concept that has gained significant attention over recent years and that could be part of the solution is the Internet of Things. It aims to connect billions of devices based on sensors accompanied of a small processing unit to the Internet with the purpose of effectively using resources in smart cities. The cohesion between smart cities and IoT was in-depth researched in multiple works [9,10], with the IoT infrastructure enabling various and massive opportunities.

The range of IoT applications is vast and not restricted only to smart cities. IoT applications have been reported to be used in healthcare, health monitoring and indoor tour guide systems [11,12]. Furthermore, the Internet of Things includes applications such as a bee activity acoustic classification system [13], a smartphone irrigation schedule tool supporting a farmer’s decision on the timing and amounts of irrigation [14] and a WSN monitoring the dosimetry of IRad operators [15]. IoT applications have evolved rapidly over the last few years and are now moving successfully to agriculture. Traditionally, sensors have been used in irrigation systems to provide information about the crop water requirements and soil temperature [16]. Other information such as data gathered from multispectral cameras or satellites are also currently used to estimate water crop needs [17,18]. Ruiz-Garcia et al. reviewed the technical and scientific state of the art of wireless sensor technologies and standards for wireless communications in the agri-food sector and concluded that in agriculture WSNs could replace the wired sensors networks in the near future [19].

This article focuses on the design and optimization of a capacitive soil humidity sensor part of a Humidity and Temperature Measurement Wireless Equipment used in the agri-tech sector. For a better understanding of the problem, the authors of this work had multiple meetings with experts from the National Institute of Agricultural Research-Development Fundulea, Romania. Conclusions were that even if existing solutions on the market already monitor soil temperature and humidity, they are either difficult to interconnect in a larger network or have a high price, making them inefficient. Following these premises, we address this issue with a cost-effective equipment that could function as a node in a WSN constituting an alternative irrigation system part of the solution to the challenges the agri-food sector is facing. Following the meetings with experts from the Institute we determined that a basic set of data required to be monitored by a WSN includes soil temperature and humidity at specific depths below the surface line and that a possible implementation should include features such as small size and energy autonomy.

Soil temperature data can be easily measured with devices already available on the market, whereas humidity needs further investigation. Soil moisture measurements were reviewed in previous works [20,21] and the limitation of each method was discussed. Soil humidity can be measured by monitoring its resistivity, a method with limited performance that raises all sort of issues such as the sensors being subjected to corrosion, which considerably reduces operating time span from years to months. The most commonly used method is the one exploiting dielectric properties of the soil with electrical capacitance of the environment changing dramatically with the proportion of water in soil. Industrial humidity sensors using capacitive methods exist on the market and have been investigated and calibrated taking into account temperature, water salinity, spatial variability of soil conditions and soil structure and texture [22,23,24]. However, capacitive sensors such as Decagon 10HS and 5TE have a relative high price making them not suitable for a low-cost equipment.

Capacitive humidity sensors work by capturing the change in the electric capacitance of soil caused by the high relative permittivity ($\epsilon_{r}$) of the water also referred as dielectric constant in this work. Measuring a changing $\epsilon_{r}$ is done by forming a capacitor with the soil dielectric, several implementations existing. The rapid development of circuit board manufacture services made PCB-made capacitive sensors extremely attractive for large scale IoT applications due to their low-price. This type of capacitive sensors captures the changing capacitance of the soil with copper traces disposed on the circuit board which benefit of the fringing effect. Copper electrodes do not have metal exposed to the soil environment and they can be coated with different epoxy solutions, the effect of corrosion being reduced to a minimum. Disadvantages of PCB-made humidity sensors include the sensitivity being reduced by the parasitic influence of the FR4 dielectric substrate and a small effective area of the sensor [25], the latter being resolved by enlarging the custom circuit board area to a desired size and arranging copper traces in an interdigitated geometry.

Capacitance-based humidity sensors have been used for years in different devices such as air conditioning systems for the control of ambient humidity or in production lines [26]. Aluminum interdigitated electrodes on a silicon substrate bonded to a PCB were used by Sweelssen et al. to monitor gas compositions and the associated methane number from gas mixtures [27,28]. Using a silicon substrate and CMOS technology comes however with a high cost and is not feasible to be implemented in agriculture applications. McIntosh and Casada manufactured a fringe field capacitive sensor for measuring the moisture content and temperature of agriculture commodities [25].

Due to the low-cost attributed with PCB-made capacitive humidity sensors we opted for using them in the equipment proposed in this work. As far as current state of the art is regarded, this type of sensors has been investigated in the past. Harris and Stonard implemented one interdigitated capacitive humidity sensor and characterized the structure as a system of three capacitors in parallel, two formed by the electrodes with the FR4 dielectric and one with the environment in which the sensor is immersed [29]. Other available on the market low-cost capacitive sensors have been experimentally characterized using the gravimetric technique [30,31]. Mizuguchi et al. also studied a fringing field capacitive sensor used to measure the water percent in the soil [32]. Even if capacitive sensors implemented on FR4 substrate have been studied in the past, there is a lack of work as far as design is regarded. To the best of our knowledge, there are no works investigating design optimization techniques for interdigitated geometries and no other study revealed how global performance of a sensor varies with the spacing or width of the PCB copper traces.

We focused this paper on both a research-oriented approach and a technical one. In the first part of this work, we start by investigating how the capacitance and sensitivity of PCB-made capacitive sensors are influenced by the aspect ratio Width/Spacing (W/S) of the copper traces and the thickness (T) of the dielectric substrate. We designed a total of 30 different capacitive sensors subjected to an in-depth analysis by both simulation and measurements. Results from this part revealed what is the best geometry to use when designing a capacitive humidity sensor to maximize performance. In the second part of this work a low-cost Humidity and Temperature Measurement Wireless Equipment design is proposed using the guidelines offered by the first part of this study.

## 2. Design and Implementation

### 2.1. Capacitive IDC Sensor

**Figure 1:**
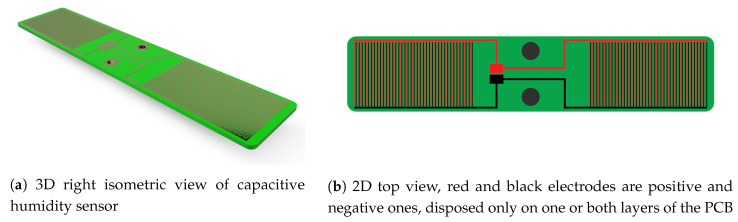
Interdigitated capacitive humidity sensor.

**Figure 2:**
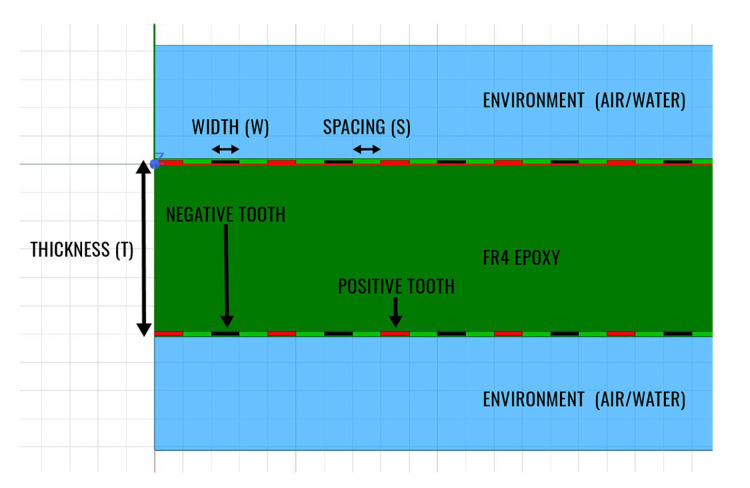
Section view of the capacitive humidity sensor. Spacing (S) and Width (W) are the parameters with the highest influence in the sensitivity of the sensor with Thickness (T) with a very small impact. The stackup is composed of soldermask, copper, FR4 epoxy, copper and soldermask.

Capacitance values above tenths of pF can be easily measured with conventional electronic circuits and provide a good signal to noise ratio. The capacitance of only two coplanar copper traces is however in the order of a few pF and thus multiple such structures must be connected in parallel to increase it. This leads to a design where multiple circuit traces are interlaced in an interdigitated (IDC) way. The so formed structure from Figure 1 with a section of the geometry visible in Figure 2 has one main feeder and tenths to hundreds of teeth connected to it, all forming one electrode of the capacitor interlaced with a second coplanar one.

In this work we designed 30 different types of PCB-made capacitive sensors to determine how the capacitance and sensitivity are influenced by the aspect ratio of the electrode teeth and thickness of the dielectric. The PCB dimensions of 4.8 by 1 inches were kept the same for all the structures regardless of their aspect ratios. Also, the effective PCB area occupied by the electrodes was identical for all the geometries, two squares of 1.55 by 0.85 inches equally distanced from the center. Applying these constrains further helps to the fair performance comparison by always referencing them to a fixed area. In the case of two-layered sensors, equivalent potential electrodes were placed one above the other on the TOP and BOTTOM copper layers to minimize the influence of the FR4 dielectric to the total capacitance.

The first lot of 18 structures named from V1-1L to V9-1L and respectively V1-2L to V9-2L designed on a fixed thickness FR4 dielectric substrate with one or two copper layers includes variations of 5, 10 and 20 MILs for W and S. A second lot of 12 sensors with 2 copper layers and fixed aspect ratios of 5/5, 10/5 and 20/5 was also investigated. In this second lot, the thickness of the PCB dielectric and how it influences the performance of the sensors was studied by varying it from 0.4 mm to 2.0 mm.

**Figure 3:**
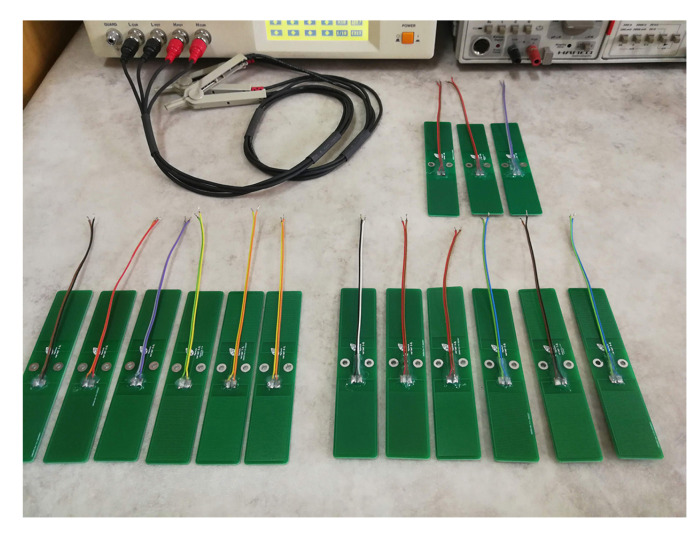
Sensors investigated in this work. 12 of them are fabricated on standard 1.6 mm thickness PCB with S and W varying from 5 to 20 MILs and 3 of them are fabricated on 0.8 mm thickness PCB with fixed aspect ratios W/S = 5/5, 10/5 and 20/5.

From the total of structures designed, 15 variations visible in Figure 3 were also manufactured. Further investigation through LCR meter measurements were considered to be of interest on this specific selection.

### 2.2. Capacitance Measurement Circuit

To develop a Humidity and Temperature Measurement Wireless Equipment the design of a low-price, low-power electronic capacitance measurement circuit was investigated. Different techniques were used in previous works such as converting the capacitance into the width of a pulse [32] or using a dedicated capacitance measurement integrated circuit [27,28], both solutions requiring expensive components in contrast to ours. Another approach of determining the capacitance is by measuring the time to charge the capacitor through a series resistor *R* under an AC square signal [29]. This charging time equal to 3·τ=3·R·C is an indirect measure of the capacitance when the value of the series resistor *R* is known. With the goal to design a battery-powered equipment, measuring a charging time in order of milliseconds could have been a real power consumption issue, hence the need for a different approach.

In the above presented approach, the half-period of the square AC signal, T/2, imposed to the capacitor is higher than the charging time, T/2>3·τ, in which case the AC voltage of the capacitors reaches the same peak voltage as the square signal after the charging is completed. However, if the half-period of the square AC signal is smaller than the charging time of the capacitor, T/2<3·τ, the peak voltage of the capacitor will never reach the maximum value and will also be dependent of the parameter of interest, C, through the formula from Equation (1). The roots of this formula are provided in Appendix A. Under these premises is also how the capacitance measurement circuit we propose operates. The main reason for following this approach is that the peak voltage can easily be converted into a DC voltage, a measure readable by a low-power MCU in just a few microseconds through an ADC conversion.

(1) $$U_{cM}=U_{i}\cdot \frac{1-e^{-\frac{T/2}{\tau }}}{1-e^{-\frac{T}{\tau }}}$$

where:

$U_{cM}$peak AC voltage of the capacitor [V]
$U_{i}$ peak AC voltage of the square signal applied to the capacitor [V]
T/2 the half-period of the signal for the 50% duty cycle signal [ns]
T   signal period [ns]
τ    time constant of the circuit, equal with the product of R and C [ns]

Results from a preliminary analysis revealed that values from 200 pF to 1200 pF were of interest to be measured by the capacitance measurement circuit, which translated into charging time intervals from 600 ns to 3600 ns if a standard 1 kΩ resistor was considered. Subsequently, the measurement circuit had to have a square signal of at least 833 kHz applied to the capacitive sensor through the series resistor in order to meet the requirement of T/2<3·τ for any capacitance value in the specified range. This lower limit of the working frequency could easily be increased by decreasing the series resistor value and thus lowering the maximum half-period of the applied square signal. Even if a frequency as high as possible is desired to minimize the effect of the losses related to the imaginary part of the complex dielectric constant [33], we opted not to go higher than 833 kHz to avoid possible signal integrity issues that could appear in the high frequency domain.

**Figure 4:**
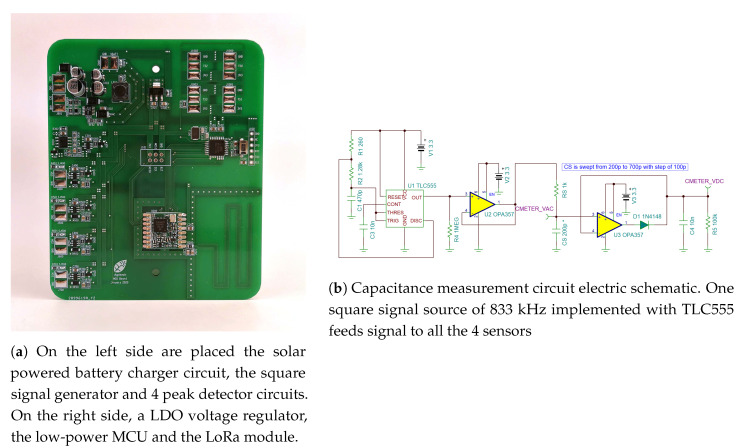
HTMWE electronic circuits, board and partial schematic.

An external square signal oscillator was preferred rather than using a PWM output from the microcontroller included in the HTMWE equipment from power optimization reasons. Designing the application as a low-power one requested the MCU to operate at a frequency of only 1 MHz, making the generation of an 833 kHz signal a real intensive task for the embedded microcode. The capacitance measurement circuit is displayed in Figure 4b with a 50% duty cycle 833 kHz square signal oscillator implemented with a TLC555, a voltage follower amplifier and a peak detector circuit, both implemented with operational amplifiers OPA357 from Texas Instruments. Even if not displayed in this electric schematic, each of the four humidity sensors has an individual peak detector circuit attached.

**Figure 5:**
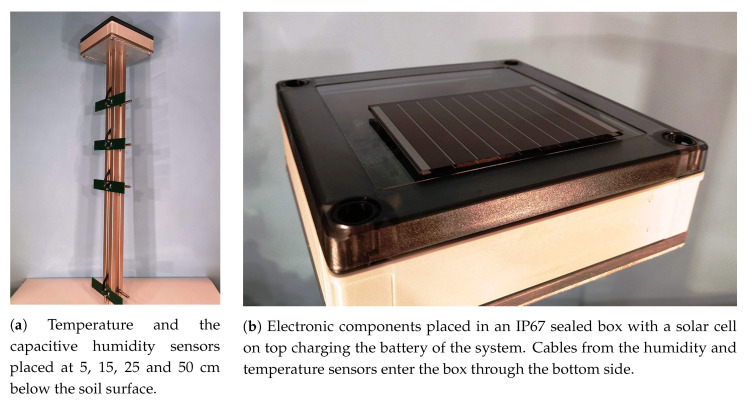
Humidity and Temperature Measurement Wireless Equipment.

The Humidity and Temperature Wireless Equipment is also equipped with a low-power Atmega 48PA microcontroller from Microchip operating at a frequency of 1 MHz and a RFM95 LoRa module from HOPERF Electronic connected with the MCU via an SPI line. The HTMWE also includes a battery charging unit from a 4.9 V Panasonic solar cell built around the integrated circuit LT3652 from Linear Technology. The circuit board from Figure 4a is equipped with all these functional blocks along with the capacitance measurement circuit and enclosed in a IP67 sealed box placed on the top of the equipment as seen in Figure 5b. For the temperature data, one-wire digital thermometer sensors DS18B20 from Maxim Integrated factory-enclosed in metallic rods are used. In Figure 5a these temperature sensors and the capacitive ones for humidity can be seen placed at depths of 5, 15, 25 and 50 cm below the soil surface. The capacitive humidity sensors are connected to the electronic circuit board with controlled impedance RG58 coaxial cable. Since the capacitance of this connection adds to the one of the sensors, a controlled capacitance cable was preferred over regular two-wires cable.

The Lithium Polymer battery of 2000 mAh which powers the Humidity and Temperature Wireless Equipment is completely charged in 40 h with a constant current of 50 mA from the solar cell charging unit. However, the main purpose of this functional block is not to fully charge the battery of the system but only to compensate for the charge drained by the rest of the system, the battery acting as a buffer during the night time when solar light is absent. In the worst-case scenario, the system has a maximum current consumption of 165.9 mA, the highest contribution of 120 mA coming from the LoRa module while transmitting data. Another 0.4 mA is consumed by the TLC555 circuit along with 4 × 7.5 mA by the OPA357 operational amplifiers. The remaining current is consumed by the Atmega 48PA MCU and the four temperature sensors. The system is designed to work in an idle mode where all functional blocks are disabled and to wake up every 30 min to collect and transmit data gathered from the environment. In this idle state the total current consumption is below 1 mA.

## 3. Materials and Methods

In this section, the methods used to simulate and measure the capacitive sensors investigated are presented. Data sets collected by either simulations or analytical approaches were correlated with measurements. The current work focuses on the characterization of a PCB-made capacitive sensor and therefore only the capacitance of this structure was investigated in different environments. A calibration of the sensor and a dependency between capacitance and the water content in the soil was not investigated, this being an important further research direction.

The quantity of interest in this part of the investigation was the capacitance between two coplanar conductors, a relatively easy calculation when the environment is homogeneous but a far more elaborate one when it becomes heterogeneous. In the particular case of a capacitive sensor, electric field lines close through three different types of dielectric materials: FR4 epoxy, soldermask and air or water. Previous work in the area of coplanar strip waveguides was done and as a result some analytical approaches exist [34,35]. These formulas separate the capacitance in a parallel circuit with each element describing only the influence of one dielectric material and then use superposition to combine the partial results. However, a simulation tool was preferred in the present work rather than analytical approaches.

ANSYS Q3D Extractor is an advanced quasi-static 3D electromagnetic field solver suited for solving problems where the electrical dimensions are shorter than λ/10 of the frequency of interest. This condition is not restrictive at all since the geometries of interest for this specific problem are well below this threshold. The reason for using this software tool among the vast suite of programs from ANSYS is that it provides results of simulation as RLCG parameters. Moreover, ANSYS Q3D Extractor has a 2D version for two-dimensional geometries which provided certain advantages in a manner described in the following paragraphs.

Three-dimensional simulations enhance the two-dimensional ones with a third dimension which brings a supplementary quantity of both additional information and processing time. Subsequently, the meshing processes is performed in a 3D way with tetrahedral elements rather than with triangular ones. If simulation time or processing resources are limitless, which is rarely the case, 3D simulations on any design should be performed since they best characterize the actual behavior of a specific geometry. However, if the desired effect which needs to be investigated does not vary along a third dimension this one can be excluded from the simulation profile resulting in a simplified 2D problem. The provided results are in the end multiplied by the third omitted dimension to obtain numerical values describing the whole 3D design.

**Figure 6:**
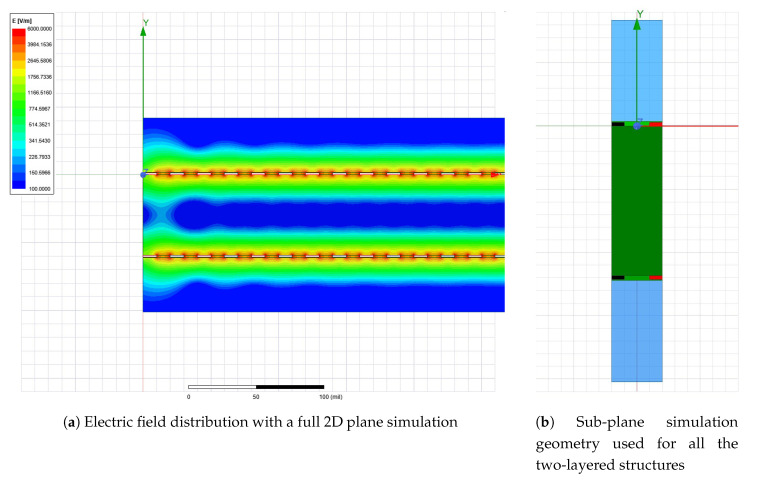
Full and reduced 2D simulation setup from ANSYS Q3D Extractor.

The main trade-off to be made in any numerical simulation is the one of accuracy vs time. Since a large number of designs were investigated in this work, each of them with different variations either of thickness or of dielectric constant of the environment, the simulation profile was optimized to obtain an accurate result with a fairly short simulation time. The PCB is in its full extend a 3D geometry, but the effect of interest, capacitance between traces can be easily reduced to a 2D problem in a plane transversal to the teeth of the two electrodes. Furthermore, the resulting plane can be reduced to only a sub-plane including half of the width of one electrode teeth, a spacing and another half of the opposite electrically charged electrode teeth. The resulting sub-plane is displayed for analysis in Figure 6b. Results are given by ANSYS 2D Extractor per unit length and therefore to obtain a total capacitance, the outputted quantity was multiplied with the length of one tooth and with the number of pairs of teeth. Because all the sensors investigated were kept to fixed dimensions regardless of their aspect ratios, the number of pairs of teeth for each sensor varies and resulted based on the geometry.

The simplifications discussed above can only be made under strict circumstances which were verified before proceeding to gathering actual simulation data. Sectioning a 3D design with a transversal plane for reducing a 3D problem to a 2D one is only valid if the measure of interest does not vary in the third omitted direction, in this case the line perpendicular to the plane. This criterion is satisfied for the structures, except of the corners where an electrode tooth also couples with the main feeder of the opposed electrically charged tooth. Considering this small coupling insignificant is a fair assumption. Another condition that had to be verified for the further reduction of the 2D plane to the sub-plane displayed in Figure 6b was that only coupling with the closest tooth is what influences the capacitance of the two electrodes. This assumption was verified by simulating the full 2D plane from Figure 6a and comparing the capacitance resulted from these two simulation setups, values being correlated with an error below 1%.

**Figure 7:**
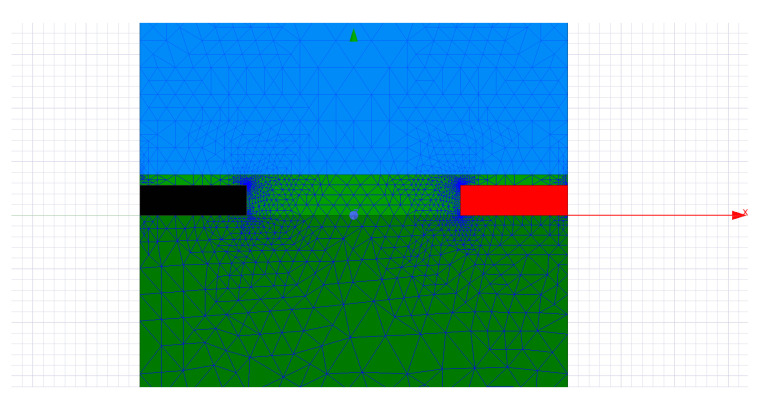
Meshing in ANSYS 2D Extractor of a section in the traces of the capacitive sensor.

Simplifying the geometry to a sub-plane from the transversal 2D plane gave us the opportunity to thoroughly investigate the capacitance between the circuit traces in all the scenarios of interest. The ANSYS 2D Extractor simulation profile was configured with an error lower than 0.01% and first order basis functions. Solution convergence was obtained in 14 to 16 iterations running on a 2.7 GHz CPU physical machine with 16 GB memory. This strict condition significantly increases the size of the generated mesh to 13,310 elements by lowering the mesh element sizes. The final mesh generated after this adaptive process visible in Figure 7 had the smallest element size of only 0.0027 MILs near the copper traces and the largest element size of 1 MIL in the FR4 dielectric. The small size of the simulation problem based on the simplifications presented provided extremely accurate results and also a small simulation time empowering us to easily explore all the design variations.

**Figure 8:**
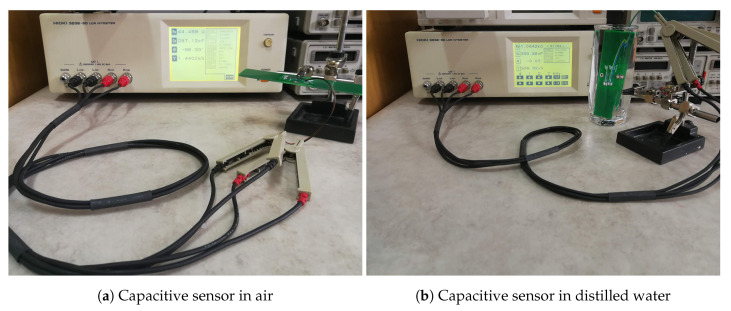
Measurement setup for capacitive sensors with a HIOKI 3532-50 LCR Meter. The DUT is connected via a quadripolar connection with a small wire fixture.

The measurement setup for the capacitance of the sensors with a HIOKI 3532-50 LCR Meter visible in Figure 8 is now discussed. Capacitance variation from its minimum value were explored by placing the sensor in plain air ($\epsilon_{r}=1$) or by immersing it in distilled water ($\epsilon_{r}=77$). This measurement setup with the capacitive structures being tested only in laboratory does not investigate the unlikely but possible corrosion effect that could take place when the sensor is left for long periods of time in a water and soil mixture.

Measurement results are presented based on a RC circuit, where R is a series or parallel resistor according to the chosen model used to characterize resistive losses. The values raging from 200 pF to 2000 pF for the capacitance resulted in a fairly small impedance ranging from 79 Ω for the 2000 pF at 1 MHz to 7957 Ω for the 200 pF at 100 kHz. A series circuit was therefore used for the measurements, this type being preferred for small impedances [36]. The frequency domain of interest from 100 kHz to 1 MHz also included the 833 kHz operating frequency of the capacitance measurement circuit.

The resonance technique was used to measure the capacitance for the investigated structures by integrating the sensors in a parallel LC circuit with an axial leaded inductor. With the value of the inductor being known and the resonance frequency experimentally determined, the capacitance could be easily obtained from the self-resonant frequency formula of an LC circuit. However, this simple expression does not take into account the series resistances of the capacitor and inductor. In the search of a more reliable characterization model, both parasitic series resistances of these components were taken into account. The complete description of this model is provided in Appendix B.

**Figure 9:**
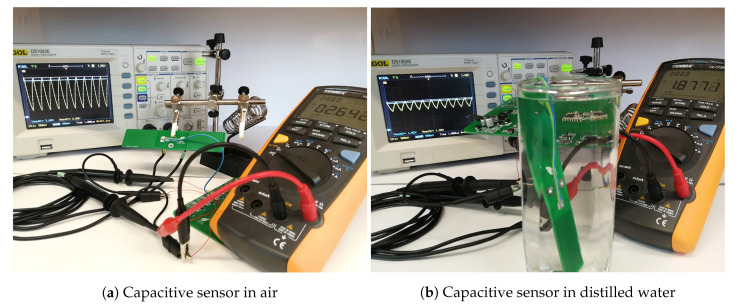
Capacitance measurement circuit investigation setup. A RIGOL DS1052E oscilloscope has CH1 connected to the AC voltage of the capacitor sensor and CH2 connected next to a TENMA 72-7730A Digital Multimeter at the DC output voltage from the peak detector circuit.

The capacitance measurement circuit was also investigated through different techniques. The simulation setup in Tina-TI from Texas Instruments and the analytical approach were broadly discussed in Section 2.2. Also a laboratory measurement with the setup displayed in Figure 9 was performed. Channel CH1 of a RIGOL DS1052E oscilloscope was connected at the capacitive sensor and CH2 together with the TENMA 72-7730A Digital Multimeter at the output of the peak circuit detector. Both the oscilloscope probes and the capacitive sensor were connected with small wire fixtures soldered to the circuit board which contributed fairly insignificant with parasitic elements to the measurement. As previously mentioned, a small error was committed by placing the input capacitance of the oscilloscope in parallel with the capacitive device under test.

## 4. Results

For all the designed structures, simulations were performed using ANSYS 2D Extractor to obtain their capacitance in different environments: air with a dielectric constant $\epsilon_{r}$ of 1 and distilled water with a $\epsilon_{r}$ swept from 10 to 77 with a step of 10. Through the simulation sweep of the dielectric constant, accurate data about the variation of capacitance from minimum to maximum was obtained. Results for the structures designed on a fixed thickness PCB are presented in Table 1, where only capacitance in air is given as absolute value of pF and the rest as increase factor (by how much the capacitance increases for a certain $\epsilon_{r}$ of the environment). Results for the structures designed on variable thickness PCB are given in Table 2, this time capacitance being given in absolute value for every experiment, since small variations are better visible in this format.

Measurements were performed on the manufactured capacitive sensors with an LCR meter with the sensors immersed in distilled water or left in plain air. The series resistance and capacitance of the sensors were measured and compared with simulated data. A lower than 10% error of the measurement regarding the simulation values was obtained. Measurements are presented in Table 3 and Table 4, where the rows grayed-out with no experimental data correspond to sensors variants simulated but not manufactured.

**Figure 10:**
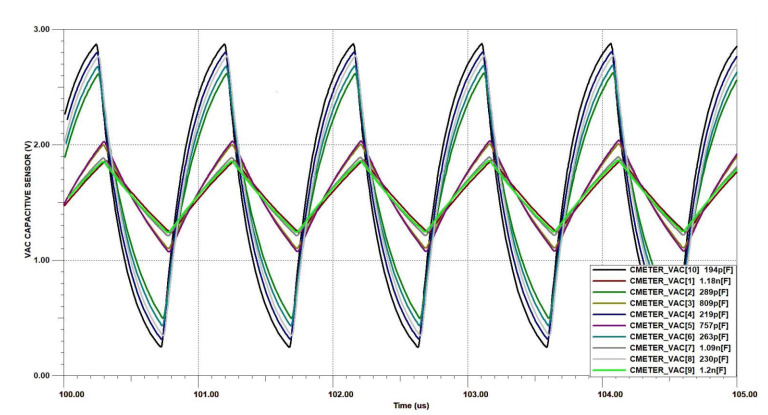
Simulated AC voltage at the capacitor sensor using TINA-TI from Texas Instruments for values corresponding to sensors V1-1L, V4-1L, V2-2L, V5-2L and V8-2L. The simulation schematic is the one from Figure 4b.

**Figure 11:**
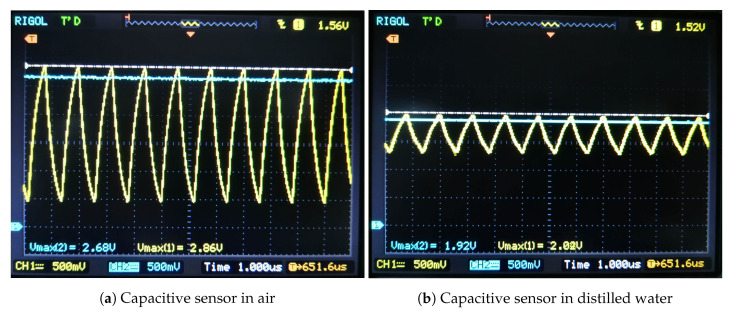
AC voltage at the capacitor sensor on CH1 and DC voltage output from the peak detector circuit on CH2. Both CH1 and CH2 are set to 500 mV/div and time base is 1 us/div. Measurement taken with a RIGOL DS1052E oscilloscope.

The capacitance measurement circuit was tested with several PCB-made sensors from the lot, all with capacitance spanning from 200 pF in plain air to 1200 pF when immersed in distilled water. The analytical data is based on Equation (1), where T/2=600 ns, T=1200 ns and $U_{i}=3.2$ V, values taken from a previous oscilloscope measurement of the AC square signal source. The simulation data resulted from TINA-TI simulator from Texas Instruments, where the capacitance $C_{s}$ from Figure 4b was swept to the corresponding values from column $C_{s-measured}$ of Table 5. The transient simulation waveforms resulted from TINA-TI are displayed in Figure 10. Lastly, measurements were performed with an oscilloscope and a digital multimeter, waveforms from oscilloscope being visible in Figure 11.

## 5. Discussion

**Figure 12:**
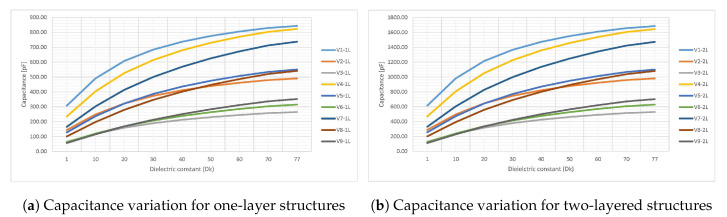
Simulation results of capacitance variation for the 18 structures when the $\epsilon_{r}$ of the environment is swept from 1 to 77. Graphs display the absolute value in pF of the capacitance obtained by multiplying the increase factor with the initial capacitance from Table 1.

**Figure 13:**
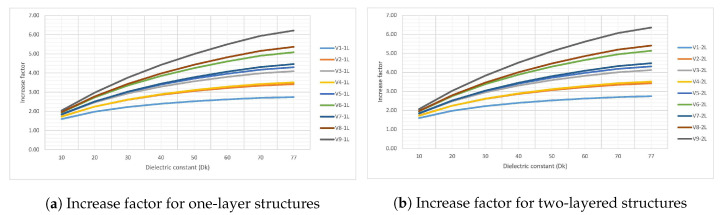
Simulation results of capacitance increase factor for the 18 structures when the $\epsilon_{r}$ of the environment is swept from 1 to 77 with value $\epsilon_{r}$ = 1 omitted from the Graph. The data from Table 1 is used.

In this section, the results, working hypotheses and their interpretation in the perspective of previous work is discussed. The data from Table 1 presented in Section 4 is here displayed as a series of graphs. Series from Figure 12a,b shows the variation of capacitance with the dielectric constant of the environment when the aspect ratio is varied and the thickness is kept fixed to the standard 1.6 mm. For further discussions, the same data from Table 1 is also displayed as variation of the increase factor in Figure 13a,b.

**Figure 14:**
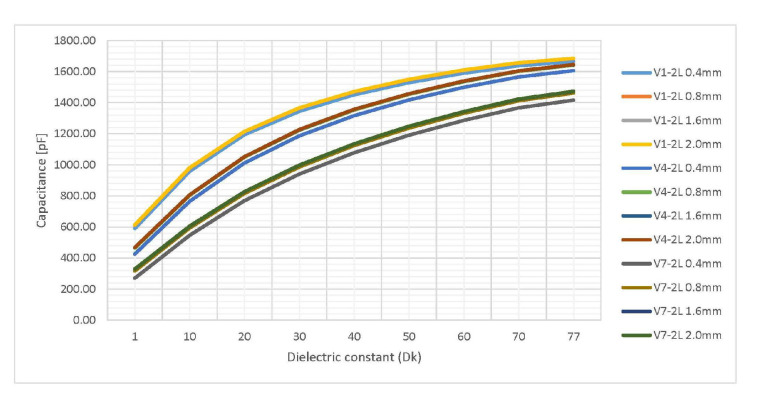
Simulation results of capacitance variation for 3 two-layered structures where the thickness is varied from 0.4 mm to 2 mm. The $\epsilon_{r}$ of the environment is swept from 1 to 77. Data from Table 2 is used.

Data from Table 2 provided in the form of a graph in Figure 14 shows the variation of capacitance with the thickness of the FR4 dielectric for fixed aspect ratio sensors V1-2L, V4-2L and V7-2L. Absolute values for the capacitance of these three sensors are displayed for thicknesses ranging from 0.4 mm to 2.0 mm. These results are later used to discuss how the thickness of the board affects the capacitance of the sensor.

The capacitive sensors performance indicators, capacitance and sensitivity, are now presented in the light of the results synthesized in graphs and tables. The constrain for all the sensors of with the electrodes laid on the same fixed rectangular circuit board area regardless of their aspect ratio allowed us now to make fair performance comparisons by referencing all the values to a fixed area. Therefore, the total capacitance of the PCB-made sensors was investigated rather than its value per only one teeth pair.

Measurements validated the initial assumptions, with the capacitance increasing when the spacing S decreases, a trend that maintains either in air, distilled water or environments with $\epsilon_{r}$ between these two extreme values. From Figure 12a,b is observed that the two-layered sensors have roughly a capacitance twice as their one-layer homologous as expected. The last parameter of interest here is the lowest possible value of the capacitance, which is around 50 pF for V3-1L, V6-1L and V9-1L or around 100 pF for V3-2L, V6-2L and V9-2L. As previously mentioned in Section 4, every sensor in the HTMWE is connected to the main electronics board with a coaxial cable of 101 pF/m which increases the capacitance with an additional value of 70 pF in the worst-case scenario. As a basic principle of measurements, the quantity desired to be measured should be higher than the additional quantity added by the fixture, reason capacitive sensors such as V3-1L, V6-1L, V9-1L, V3-2L, V6-2L and V9-2L were not considered to be having good performance in this topic of interest.

So far, only the capacitance of the sensor was investigated, but another important figure of interest is its sensitivity: how responsive it is to a small change in the dielectric constant of the environment. For this aspect, the data from Figure 13a,b is discussed. The increase factor in capacitance has the same trend for both the one and two-layered structures, an expected result since the capacitance roughly doubles when the number of copper layers doubles. The structures with the highest sensitivity with an increase factor of almost 5 from plain air to fully immersed in water, V9-1L, V8-1L, V6-1L are exactly the sensors with a low minimum capacitance, well below the average. This is a startling result in the light of previous work [32] which stated that the best geometry is one with the electrodes as close as possible to increase capacitance without considering the sensitivity of the sensor. If for example the highest capacitance sensor from the lot, V1-2L, is taken into account, a worst possible sensitivity is obtained because it increases its capacitance by a factor of only 2.5 when immersed in water.

**Figure 15:**
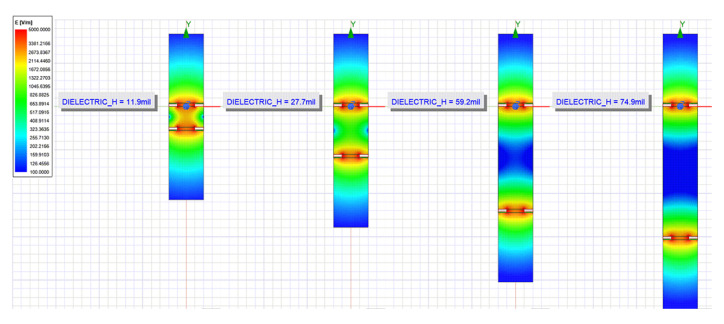
Electric Field distribution between the traces of the capacitive sensors for different thicknesses of the FR4 dielectric. One trace is positively charged and the other is negatively charged.

Thierauf stated that higher dielectric thickness leads to the electric field density inside the board lowering, resulting in more electric field lines closing outside the board through the environment, which could lead to a higher capacitance sensor caused by the high dielectric constant of the environment [37]. Data from Figure 14 where variation of the thickness of the dielectric substrate is presented shows a slightly increase in capacitance for thicker FR4 dielectric sensors. This is due to the electric field being disposed with a lower density inside the board when the dielectric thickness increases as observed through the electric fields plot exported from ANSYS 2D Extractor in Figure 15. However, this slight increase in capacitance is not enough to justify the two to three times higher production cost for using thicker FR4 dielectrics.

After this in-depth analysis of aspect ratio and thickness, the sensor named V8-2L was found to have the best performance: good sensitive with capacitance increasing by a factor of 5 when immersed in water and a high enough minimum value of 199 pF when placed in plain air. This trade-off between sensitivity and capacitance was considered the best of all, only V9-2L surpassing these figures of performance but with the price of a minimum capacitance of only 110 pF in air. As mentioned before, using dielectric thicknesses other than the standard 1.6 mm was not proved to have a high impact. The sensor V8-2L was also used in the Humidity and Temperature Measurement Wireless Equipment.

Measurement of the capacitance for the sensors synthesized in Table 3 and Table 4 are now discussed with regard to the errors for the measured values to the simulated ones. The reason for considering the simulated ones as ideal is that special consideration was taken into elaboration the ANSYS 2D Extractor model, a topic broadly elaborated in Section 3. Since a simple series RC circuit was used to describe the capacitor, the parasitic resistance was expected to have a reduced value. Measurements have shown that this resistance drops even more when the sensor is immersed in distilled water. Behzadi pour and Golnabi proved that when the electric coupling increases, such as the case of electrodes immersed in a high dielectric constant environment as water, the series resistance should decrease [38].

To investigate the capacitance measurement circuit 5 capacitive sensors of values ranging from 200 pF to 1200 pF were selected. Measurements of the output signal were performed with a RIGOL DS1052E oscilloscope and a TENMA 72-7730A Digital Multimeter and the results were noted in Table 5. The measured values of AC and subsequently DC voltage are lower than the ones simulated in TINA-TI from Texas Instruments because they correspond to a higher capacitance than the one of the sensor. This higher capacitance was caused by the addition of 18 pF ± 3 pF from the input of the oscilloscope connected in parallel with the sensor.

One effect not investigated in this work and that leads to further research directions is the variation the capacitance of the sensors has with temperature, all measurements and simulations being performed in plain air and water at room temperature. Mizuguchi et al. investigated how temperature influences the capacitance of an interdigitated capacitive sensor, with results showing that capacitance drops with temperature from 675 pF at 0 ∘C to 650 pF at 20 ∘C and to 620 pF at 45 ∘C for a specific geometry and aspect ratio [32]. They also calculated the error committed by only measuring the capacitance of the sensor at room temperature at around 20 ∘C which leads to an error of up to 5% from 0 ∘C to 45 ∘C. This error could easily be corrected inside the embedded software of the microcontroller from the HTMWE, since temperature is already an input data available to the equipment via sensors. Finally, dielectric constant of air is less dependent of temperature and can therefore be considered constant.

## 6. Conclusions

The findings of this work resulted from both simulations and measurements demonstrate that interdigitated capacitive structures have potential as low-cost soil moisture sensors. By investigating various geometries, this work provides an in-depth comparative study of various aspect ratio capacitive sensors. In contrast with previous work, this investigation revealed that a high capacitance comes with the cost of a low sensitivity and that for a higher-accuracy lower-capacitance sensor should be used.

All the investigations of this work were performed only in a laboratory environment at room temperature, with the sensors being analyzed in two different cases, first with the structures placed in plain air and secondly with them immersed in distilled water. Therefore, the full specter of values for the capacitance was determined. Performing measurements only at room temperature is considered only a systematic error which can easily be corrected with several formulas documented in the current state of the art and referenced in this research. Also, our investigation setup gives no information about the unlikely but possible corrosion of the sensor when placed in a real soil and water mixture for long periods of time.

In the last part of this work, a Humidity and Temperature Measurement Wireless Equipment developed to gather temperature and humidity soil data from different soil depths using the finding of the first part of this work was designed. The HTMWE is developed to operate as a node in a Wireless Sensors Network, allows the future development of smart agriculture integrated solutions and is suitable for monitoring soil parameters by gathering data that can be later used in predictions and modeling.

Further research of development include optimization for low power of the HTMWE and improved data processing routines. Also, the error produced by the characterization of the sensor only at the room temperature will be corrected in software. Nevertheless, future testing with the HTMWE will take place in a relevant environment, with sensors calibrated by immersion in a soil and water mixture. The correlation between the capacitance of the sensor and the soil humidity will be performed using the gravimetric water content principle documented in a future paper.

## Figures and Tables

**Table 1:** Simulation and measurement results for the structures designed on a fixed thickness PCB. The environment is air ($\epsilon_{r}=1$) or distilled water ($\epsilon_{r}$ swept from 10 to 77). Capacitance is listed as absolute value in pF only for air and as increase factor for the rest of the cases.

Dielectric Constant			1	10	20	30	40	50	60	70	77
Sensor	W [MIL]	S [MIL]	C [pF]	Factor	Factor	Factor	Factor	Factor	Factor	Factor	Factor
V1-1L	5	5	307.18	1.60	1.98	2.22	2.40	2.52	2.62	2.70	2.74
V2-1L		10	143.57	1.73	2.25	2.60	2.86	3.06	3.21	3.34	3.41
V3-1L		20	64.63	1.83	2.47	2.93	3.29	3.57	3.79	3.98	4.09
V4-1L	10	5	234.58	1.72	2.24	2.61	2.89	3.11	3.28	3.42	3.51
V5-1L		10	128.00	1.85	2.51	3.01	3.40	3.71	3.96	4.17	4.30
V6-1L		20	61.97	1.94	2.73	3.34	3.84	4.26	4.60	4.90	5.08
V7-1L	20	5	165.13	1.83	2.51	3.02	3.44	3.78	4.07	4.31	4.46
V8-1L		10	100.87	1.96	2.78	3.43	3.97	4.43	4.82	5.15	5.36
V9-1L		20	56.70	2.04	2.97	3.75	4.42	4.99	5.49	5.93	6.21
V1-2L	5	5	613.81	1.60	1.98	2.23	2.40	2.52	2.62	2.70	2.75
V2-2L		10	286.50	1.73	2.25	2.60	2.86	3.06	3.22	3.35	3.42
V3-2L		20	128.33	1.84	2.48	2.95	3.31	3.59	3.82	4.01	4.12
V4-2L	10	5	468.40	1.72	2.25	2.62	2.90	3.11	3.28	3.43	3.51
V5-2L		10	255.06	1.85	2.52	3.02	3.41	3.72	3.97	4.18	4.31
V6-2L		20	122.47	1.95	2.75	3.38	3.88	4.30	4.65	4.95	5.13
V7-2L	20	5	328.80	1.84	2.51	3.03	3.45	3.79	4.08	4.33	4.48
V8-2L		10	199.87	1.97	2.79	3.46	4.00	4.46	4.85	5.19	5.41
V9-2L		20	110.52	2.06	3.03	3.83	4.51	5.10	5.61	6.07	6.35

**Table 2:** Simulation and measurement results for the structures designed on variable thickness PCB. The environment is air ($\epsilon_{r}=1$) or distilled water ($\epsilon_{r}$ swept from 10 to 77). Capacitance is listed as absolute value in pF in all the cases.

Dielectric Constant				1	10	20	30	40	50	60	70	77
Sensor	W [MIL]	S [MIL]	T [mm]	C [pF]	C [pF]	C [pF]	C [pF]	C [pF]	C [pF]	C [pF]	C [pF]	C [pF]
V1-2L	5	5	0.4	589.98	958.29	1193.55	1345.06	1451.02	1529.28	1589.50	1637.28	1665.24
			0.8	611.76	978.73	1213.34	1364.51	1470.14	1548.25	1608.30	1655.95	1683.86
			1.6	613.81	980.39	1214.88	1365.89	1471.54	1549.60	1609.63	1657.28	1685.19
			2	614.04	980.55	1215.03	1366.07	1471.67	1549.70	1609.73	1657.38	1685.27
V4-2L	10	5	0.4	425.71	764.96	1011.47	1186.00	1316.48	1417.98	1499.23	1565.77	1605.60
			0.8	464.14	802.47	1048.50	1222.57	1352.84	1454.14	1535.24	1601.63	1641.40
			1.6	468.40	806.35	1052.19	1226.13	1356.31	1457.51	1538.52	1604.90	1644.63
			2	468.69	806.60	1052.44	1226.37	1356.52	1457.75	1538.72	1605.03	1644.77
V7-2L	20	5	0.4	270.25	546.26	768.84	940.38	1077.84	1190.86	1285.61	1366.26	1415.97
			0.8	317.29	593.05	815.41	986.82	1124.10	1237.01	1331.67	1412.26	1461.92
			1.6	328.80	604.25	826.45	997.68	1134.86	1247.67	1342.25	1422.77	1472.39
			2	329.44	604.86	827.05	998.26	1135.43	1248.23	1342.77	1423.30	1472.92

**Table 3:** Simulation and measurement results for the structures designed on a fixed thickness PCB. The environment is air ($\epsilon_{r}=1$) or distilled water ($\epsilon_{r}=77$). Variants with grayed-out rows were not manufactured.

			Simulation		Measurement				Error	
Sensor	W [MIL]	S [MIL]	$C_{\mathrm{AIR}}\left[\mathrm{pF}\right]$	$C_{\mathrm{WATER}}\left[\mathrm{pF}\right]$	$R_{s\mathrm{AIR}}[\Omega ]$	$C_{s\mathrm{AIR}}\left[\mathrm{pF}\right]$	$R_{s\mathrm{WATER}}[\Omega ]$	$C_{s\mathrm{WATER}}\left[\mathrm{pF}\right]$	AIR [%]	WATER [%]
V1-1L	5	5	307.18	842.67	46.22	289.34	9.57	809.76	5.81%	3.91%
V2-1L		10	143.57	490.17	61.48	129.73	26.54	536.87	9.64%	9.53%
V3-1L		20	64.63	264.61						
V4-1L	10	5	234.58	822.51	51.19	219.21	14.09	757.60	6.55%	7.89%
V5-1L		10	128.00	550.26	63.12	118.83	28.71	605.78	7.17%	10.09%
V6-1L		20	61.97	314.81						
V7-1L	20	5	165.13	736.70	55.54	149.87	23.48	694.62	9.24%	5.71%
V8-1L		10	100.87	540.91	69.98	97.55	40.25	574.12	3.29%	6.14%
V9-1L		20	56.70	352.25						
V1-2L	5	5	613.81	1685.19	40.45	563.12	10.61	1632.41	8.26%	3.13%
V2-2L		10	286.50	980.07	46.45	263.12	9.35	1086.00	8.16%	10.81%
V3-2L		20	128.33	528.75						
V4-2L	10	5	468.40	1644.63	41.47	423.76	9.36	1694.01	9.53%	3.00%
V5-2L		10	255.06	1099.96	46.49	230.58	15.85	1199.90	9.60%	9.09%
V6-2L		20	122.47	628.69						
V7-2L	20	5	328.80	1472.39	45.20	302.76	21.51	1597.81	7.92%	8.52%
V8-2L		10	199.87	1080.37	48.60	194.26	35.13	1185.11	2.80%	9.69%
V9-2L		20	110.52	702.11						

**Table 4:** Simulation and measurement results for the structures designed on variable thickness PCB. The environment is air ($\epsilon_{r}=1$) or distilled water ($\epsilon_{r}=77$). Variants with grayed-out rows were not manufactured.

				Simulation		Measurement				Error	
Sensor	W [MIL]	S [MIL]	T [mm]	$C_{\mathrm{AIR}}\left[\mathrm{pF}\right]$	$C_{\mathrm{WATER}}\left[\mathrm{pF}\right]$	$R_{s\mathrm{AIR}}[\Omega ]$	$C_{s\mathrm{AIR}}\left[\mathrm{pF}\right]$	$R_{s\mathrm{WATER}}[\Omega ]$	$C_{s\mathrm{WATER}}\left[\mathrm{pF}\right]$	AIR [%]	WATER [%]
V1-2L	5.00	5.00	0.40	589.98	1665.24						
			0.80	611.76	1683.86	41.69	564.76	9.12	1870.90	8.76%	9.99%
			1.60	613.81	1685.19	40.45	563.12	10.61	1632.41	9.02%	4.26%
			2.00	614.04	1685.27						
V4-2L	10.00	5.00	0.40	425.71	1605.60						
			0.80	464.14	1641.40	43.16	432.09	17.91	1758.21	7.99%	5.87%
			1.60	468.40	1644.63	41.47	423.76	9.36	1694.01	10.58%	1.80%
			2.00	468.69	1644.77						
V7-2L	20.00	5.00	0.40	270.25	1415.97						
			0.80	317.29	1461.92	46.60	298.43	23.98	1559.21	7.04%	5.41%
			1.60	328.80	1472.39	45.20	302.76	21.51	1597.81	8.99%	7.26%
			2.00	329.44	1472.92						

**Table 5:** Analytical, simulation and measurement results for the capacitance measurement circuit. The peak AC voltage at the capacitor sensor and the DC value outputted from the peak detector circuit are investigated.

			Analytical	Simulation		Measurement		
Sensor	Environment	$C_{s-\mathrm{measured}}\left[\mathrm{pF}\right]$	$V_{\mathrm{peak}}\left[V\right]$	$V_{\mathrm{peak}}\left[V\right]$	$V_{\mathrm{DC}}\left[V\right]$	$V_{\mathrm{peak}\mathrm{oscilloscope}}\left[V\right]$	$V_{\mathrm{DC}\mathrm{oscilloscope}}\left[V\right]$	$V_{\mathrm{DC}\mathrm{multimeter}}\left[V\right]$
V1-1L	AIR	289.34	2.84	2.83	2.67	2.70	2.58	2.53
	WATER	809.76	2.17	2.16	2.08	2.12	2.04	1.99
V4-1L	AIR	219.21	3.01	2.97	2.72	2.86	2.70	2.63
	WATER	757.60	2.20	2.19	2.12	2.18	2.08	2.05
V2-2L	AIR	263.12	2.90	2.88	2.69	2.74	2.60	2.57
	WATER	1086.11	2.03	2.01	1.95	2.04	1.96	1.92
V5-2L	AIR	230.58	2.98	2.95	2.72	2.78	2.66	2.60
	WATER	1199.90	1.99	1.97	1.89	1.98	1.92	1.88
V8-2L	AIR	194.26	3.06	3.02	2.74	2.86	2.68	2.64
	WATER	1185.11	2.00	1.97	1.91	1.98	1.92	1.88

**Figure A1:**
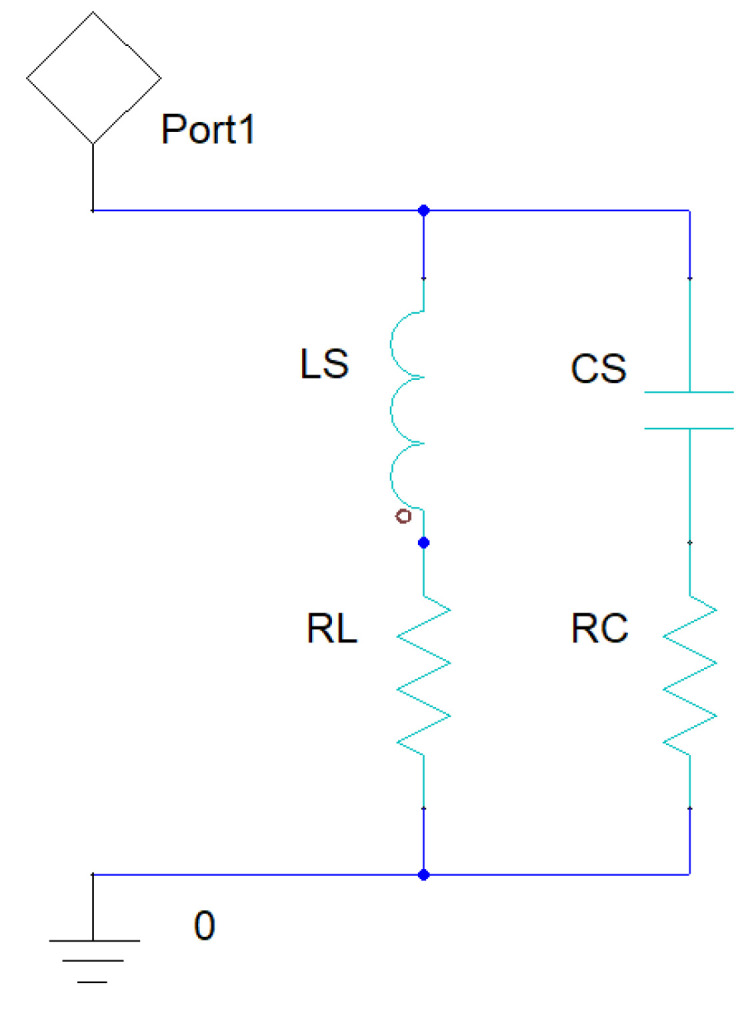
LC parallel circuit characterization of measurement setup at LCR meter used for measuring series capacitance and resistance for sensors.

## Abbreviations

The following abbreviations are used in this manuscript:

ADC	Analog to Digital Convertor
HTMWE	Humidity and Temperature Measurement Wireless Equipment
IDC	Interdigitated
LCR	(L) Inductance (C) Capacitance and (R) resistance
LoRa	Long Range
MIL	One Thousandth of an Inch
MCU	microcontroller
PCB	Printed Circuit Board
UN	United Nations
WQMCM	Water Quality Monitoring, Control and Management
WSN	Wireless Sensors Network

## Appendix A. Peak Voltage at Capacitor under Square Signal with Half-Period Lower than the Time Constant of the Circuit

In this analysis an AC square signal imposed to a capacitor (C) through a series resistor (R) is considered. In the following, some notations are made: *T* is the period of the signal, T/2 is the half-period of the signal, $U_{i}$ is the peak AC voltage of the signal and τ is the time constant of the circuit equal with the product of R and C. In the transitory time slot at the beginning of the operation of the circuit, the capacitance charges and discharges, the discharge not being fully allowed to take place by the small time slot of T/2 leading to a higher and higher voltage on the capacitor. After several periods passes, the circuit reaches a quasi-stationary state where the difference from the lowest voltage named $U_{cm}$ to the highest one named $U_{cM}$ at the capacitor is constant from one period to another. The expressions for these voltages after equilibrium state is reached in the circuit, so after a long enough period of time has passed, are now investigated.

In the positive half-period of the signal, the capacitance charges from $U_{cm}$ to $U_{cM}$, whose value is given by Equation (A1), while in the next half-period of the signal the capacitance discharges down to $U_{cm}$ following Equation (A2). By replacing Equation (A2) in Equations (A1) and (A3) is obtained. Finally, from Equation (A3), the relation between $U_{cM}$ and the capacitance results in Equation (A4) which is also presented in Section 4 of this work under the name of Equation (1).

(A1) $$U_{cM}=U_{i}+\left(U_{cm}-U_{i}\right)\cdot e^{-\frac{T/2}{\tau }}=U_{i}\cdot \left(1-e^{-\frac{T/2}{\tau }}\right)+U_{cm}\cdot e^{-\frac{T/2}{\tau }}$$

(A2) $$U_{cm}=U_{cM}\cdot e^{-\frac{T/2}{\tau }}$$

(A3) $$U_{cM}=U_{i}\cdot \left(1-e^{-\frac{T/2}{\tau }}\right)+U_{cM}\cdot e^{-\frac{T/2}{\tau }}\cdot e^{-\frac{T/2}{\tau }}=U_{i}\cdot \left(1-e^{-\frac{T/2}{\tau }}\right)+U_{cM}\cdot e^{-\frac{T}{\tau }}$$

(A4) $$U_{cM}=U_{i}\frac{1-e^{-\frac{T/2}{\tau }}}{1-e^{-\frac{T}{\tau }}}$$

## Appendix B. Characterization Model of LC Circuit Used in the Resonance Method at the Measurements Performed with the LCR Meter

By the same remarks made in Section 3 about the series model being preferred for small impedances, the inductor placed in parallel with the capacitor in the resonance method is characterized using this model. The inductor was also measured in the frequency domain of interest from 100 kHz to 1 MHz with the same LCR meter and obtained a series inductance of 112 μH and a series resistance of 45.6 Ω being almost constant in this interval. These are the values used in the following equations for *L* and $R_{L}$.

The circuit developed for the resonance measurement method is displayed in Figure A1. In Equation (A5) an expression for the admittance of this parallel circuit is developed where the notation from (A6) was made (*q* has no relation with the quality factor of an inductor). By imposing the condition of resonance on the expression from Equation (A5), the imaginary part of it is forced to be null. Therefore, the expression for $C_{S}$ is extracted in Equation (A7) which is still dependent of *q*. The unknown *q* can be obtained from the value of $|Y_{r}|$ from Equation (A8).

The measured figures from the LCR meter are $|Y_{r}|$ and $\omega_{r}$. With these two values, from Equation (A8) a value for *q* is obtained and with that introduced in Equation (A7) the series capacitance is also obtained. Once *q* and C are known, the series resistance $R_{c}$ can also easily be obtained from Equation (A6).

(A5) $$Y=\frac{R_{L}}{R_{L}^{2}+{\left(\omega \cdot L_{S}\right)}^{2}}+\frac{\omega^{2}\cdot q\cdot C_{S}}{1+{\left(\omega \cdot q\right)}^{2}}+j\cdot \left[\frac{\omega \cdot C_{S}}{1+{\left(\omega \cdot q\right)}^{2}}-\frac{\omega \cdot L_{S}}{R_{L}^{2}+{\left(\omega \cdot L_{S}\right)}^{2}}\right]$$

(A6) $$q=R_{C}\cdot C_{S}$$

(A7) $$\omega =\omega_{r},Im\left(Y\right)=0\Rightarrow C_{S}=\frac{L_{S}\cdot \left[1+{\left(\omega_{r}\cdot q\right)}^{2}\right]}{R_{L}^{2}+{\left(\omega_{r}\cdot L_{S}\right)}^{2}}$$

(A8) $$\left|Y_{r}\right|=Re\left(Y\right)\Rightarrow q=\frac{\left[|Y_{r}|-\frac{R_{L}}{R_{L}^{2}+{\left(\omega_{r}\cdot L_{S}\right)}^{2}}\right]\cdot \left[R_{L}^{2}+{\left(\omega_{r}\cdot L_{S}\right)}^{2}\right]}{\omega_{r}^{2}\cdot L_{S}}$$
